# Pre- and Post-Pandemic Religiosity and Mental Health Outcomes: A Prospective Study

**DOI:** 10.3390/ijerph20116002

**Published:** 2023-05-30

**Authors:** Connie Svob, Eleanor Murphy, Priya J. Wickramaratne, Marc J. Gameroff, Ardesheer Talati, Milenna T. van Dijk, Tenzin Yangchen, Myrna M. Weissman

**Affiliations:** 1 Department of Psychiatry, Vagelos College of Physicians and Surgeons, Columbia University, New York, NY 10032, USA; 2 Division of Translational Epidemiology, New York State Psychiatric Institute, New York, NY 10032, USA; 3 Department of Epidemiology, Mailman School of Public Health, Columbia University, New York, NY 10032, USA

**Keywords:** pandemic, religion, depression, anxiety, psychology, longitudinal, risk

## Abstract

Relatively few studies have prospectively examined the effects of known protective factors, such as religion, on pandemic-related outcomes. The aim of this study was to evaluate the pre- and post-pandemic trajectories and psychological effects of religious beliefs and religious attendance. Male and female adults (*N* = 189) reported their beliefs in religious importance (*RI*) and their religious attendance (*RA*) both before (*T1*) and after (*T2*) the pandemic’s onset. Descriptive and regression analyses were used to track *RI* and *RA* from *T1* to *T2* and to test their effects on psychological outcomes at T1 and T2. The participants who reported a decrease in religious importance and attendance were greater in number than those who reported an increase, with *RI* (36.5% vs. 5.3%) and *RA* (34.4% vs. 4.8%). The individuals with decreased *RI* were less likely to know someone who had died from COVID-19 (O.R. =0.4, *p* = 0.027). The T1 *RI* predicted overall social adjustment (*p* < 0.05) and lower suicidal ideation (*p* = 0.05). The *T2 RI* was associated with lower suicidal ideation (*p* < 0.05). The online *RA* (*T2*) was associated with lower depression (*p* < 0.05) and lower anxiety (*p* < 0.05). Further research is needed to evaluate the mechanisms driving decreases in religiosity during pandemics. Religious beliefs and online religious attendance were beneficial during the pandemic, which bodes well for the use of telemedicine in therapeutic approaches.

## 1. Introduction

The practice of religion in times of crisis remains a fundamental part of the way in which people make sense of how sickness, death, and suffering fit into a larger scheme of life. Religious coping was evident during the height of the recent COVID-19 pandemic and generated considerable scientific interest in the effects of religion on psychological wellbeing [1,2,3,4]. According to a research study on social trends during the pandemic [5], over half of US adults (55%) prayed for an end to the spread of coronavirus. Furthermore, most of the Americans who prayed daily (86%), including 73% of US Christians and nearly a quarter of individuals who claimed no religious affiliation at all, reportedly took up prayer as a response to the outbreak [5].

The unprecedented stressors associated with COVID-19, including the illness and death of loved ones, coworkers, and acquaintances, fear of becoming ill or dying from COVID-19, and the unpredictability of the pandemic produced a marked behavioral response that involved religious coping both globally and in the United States (US) [6,7,8,9,10,11,12,13]. In addition, self-isolation and shelter-in-place mandates, the re-arrangement of work environments and schedules, and the shutting down of social and recreational venues also had potentially serious implications for individuals at risk for mental disorders [14,15,16,17].

This emergent body of research on the relationships among religiosity, mental health, and the COVID-19 pandemic has highlighted two major indices of religiosity–intrinsic religious beliefs and religious attendance–both of which have lent themselves to scientific investigation during the pandemic. At this point, when access to mental health care and religious services were challenged, it was an opportune moment to better understand the role of personal religious beliefs and attendance in mental health during the pandemic.

Several studies investigated the role of religious beliefs and attendance as potential protective factors in mental health during the COVID-19 pandemic [9,18,19,20,21]. However, most relied on retrospective reports of pre-pandemic religiosity, creating a need for prospective studies on the effects of religiosity on mental health, as well as the impact of the pandemic on religious beliefs and practices. This study leveraged a longitudinal study of families at high or low risk for depression [22,23,24,25] to prospectively characterize the trajectory of religious beliefs and religious attendance before and during the pandemic, and to examine the impact of belief in religious importance (*RI*) and religious service attendance (*RA*) on mental health outcomes during the pandemic. Using participants from a longitudinal high-risk project and prior to the pandemic, a published study found that among those at high risk for depression, belief in the importance of religion was protective against their depression and suicidal behavior in their offspring [26,27,28]. Those analyses were mainly limited within a cross-sectional scope, and a prominent stressor experienced by all the participants was lacking. 

In the wake of the pandemic and without these two limitations, the present study was conducted. Its primary was aims were to characterize the trajectories of belief in religious importance (*RI*) and religious attendance (*RA*) at *T1* (pre-pandemic) and *T2* (post-pandemic onset), as well as to examine the effects of *RI* and *RA* on psychiatric symptoms and psychological wellbeing after the onset of the COVID-19 pandemic. Because a decline in in-person religious attendance due to social distancing and lockdown procedures during the pandemic was anticipated, an item evaluating online or virtual religious attendance was included. Accordingly, the hypotheses were that *RI* and *RA* (in-person or online) at *T2* would be associated with fewer depressive and anxiety symptoms, lower suicidality, and greater psychological well-being at *T2*. The potential moderation of factors such as gender, age, MDD-risk status, and psychiatric history was explored. Taken together, in this natural experiment, in which all the participants were exposed to the same stressor to varying degrees, the role of religiosity in the resilience of individuals at high and low risk for depression was more accurately determined. The questions asked of the respondents used religiosity and spirituality interchangeably and, therefore, in this study, we use the two terms simultaneously. However, we are aware that in many other contexts, religiosity and spirituality represent distinct concepts and, hence, different interpretations of their effects may arise.

## 2. Materials and Methods

### 2.1. Data Source

The study’s data came from the high-risk depression study, which is an ongoing 40-year multi-generational project comprising families at high and low risk for depression [22,23,24,25]. The objective of the longitudinal study was to evaluate risk factors and their familial and cross-generation transmission for individuals at high or low risk of major depressive disorder (MDD). The original participants comprised G1 (probands), and then their offspring (G2) were assessed [23], followed by their grand-offspring (G3) [25]. More recently, new data were collected on great-grand offspring (G4) [24]. The sample was deeply characterized through multiple clinical interviews, psychiatric and psychological symptom measures (including measures of depression, anxiety disorders, substance abuse, and suicidality), and childhood histories, as well as data from electrophysiology (EEG), magnetic resonance imaging (MRI), and DNA. The most recent data prior to the pandemic’s onset were collected over three years (2017–2020) preceding the COVID-19 outbreak in the US (*T1*) and included questionnaires on religious beliefs and practices. Therefore, we were in a unique position to track the changes in religious beliefs and attendance during the pandemic. Additionally, we were able to examine the effects of religious beliefs and attendance on post-COVID-19 mental health (*T2*), while controlling for pre-COVID-19 levels of religiosity, affective disorders, suicidality, and psychological wellbeing. 

### 2.2. Sample

**Figure 1:**
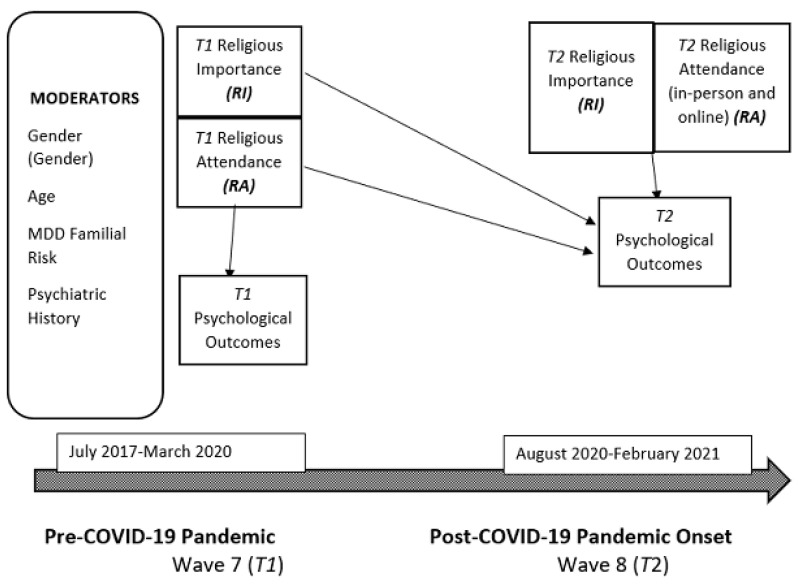
Effects of religiosity on mental health outcomes: religious importance (*RI*), religious attendance (*RA*), and psychological outcomes before (*T1*) and after (*T2*) COVID-19 onset.

In total, 249 individuals from the high-risk longitudinal cohort completed assessments at the pre-pandemic wave (*T1*), which spanned 2.5 years from 2017 to April 2020. Out of this group, 204 individuals responded to an online survey during the COVID-19 pandemic (*T2*), from August 2020 to February 2021, yielding a *T2* response rate of 82%. The current study’s participants came from G2 and G3 and ranged in age from 19–69 years. The data were collected and stored using the HIPAA-compliant and NYSPI IRB-approved software [29]. The demographic and clinical characteristics of this online COVID-19 sample and the study procedures are detailed extensively elsewhere [30]. Seven individuals completed their *T1* assessments after the onset of the COVID-19 pandemic and were consequently dropped from our analyses. For analyses involving religious importance at *T1*, we omitted an additional 8 cases due to missing *T1* data on religious importance, leaving a *T1* religious importance sample of *N* = 189. For analyses involving religious attendance at *T1*, we omitted an additional 10 cases for missing *T1* data on religious attendance, leaving a *T1* religious attendance sample of *N* = 187. The omitted cases did not differ significantly from the analytic samples on demographic characteristics or psychiatric symptoms. The timeline and assessment model for this study are shown in Figure 1.

### 2.3. Measures

Clinical measures and religiosity predictors were items from Schedule for Affective Disorders and Schizophrenia (SADS) [31], lifetime and truncated versions. Psychiatric history was based on SADS DSM-based diagnoses, with best-estimated reliability procedures from experienced, doctoral-level clinicians [32]. Items on beliefs about importance of religion/spirituality and religious attendance were included in the SADS Demographics section at *T1*, and in the COVID survey at *T2*. We did not ask participants’ religious affiliation in the COVID survey, but previous assessments from the structured interviews showed that the majority identified as Roman Catholic (>80%). Therefore, we did not attempt to evaluate religious beliefs or attendance based on religious denomination.

### 2.4. Religious Importance (RI)

We used one item on beliefs about religious importance measured at *T1* and *T2*. Respondents were asked: “How important to you is religion or spirituality?” Responses were scored on a Likert scale and coded into the following four categories: 0 = not at all important, 1 = slightly important, 2 = moderately important, 3 = highly important. Thus, scores on *RI* ranged from a low of 0 to a high of 3. 

### 2.5. Religious Attendance (RA)

In-person (*T1* & *T2*): Respondents were asked about their current religious attendance patterns using the following item: How often, if at all, do you attend church, synagogue, or other religious or spiritual services? Responses were coded into the following four categories: 0 = never, 1 = less than twice a year, 2 = about once a month, and 3 = once a week or more. Scores on *RA* ranged from a low of 0 to a high of 3. 

Online Religious Attendance (*T2* only): In addition, participants were asked about their online access to religious services during the pandemic (*T2*), and responses were as follows: 0 = no/do not attend church, 1 = less often than usual, 2 = as often as/more often than usual. Scores on online *RA* ranged from 0 to 2.

### 2.6. Mental Health Outcomes

Psychiatric and psychological symptoms were assessed using the Personal Health Questionnaire-9 item (PHQ-9) [33], Inventory of Depression and Anxiety-Version 2 (IDASII) [34,35], and the Social Adjustment Scale-Short Form (SASSR) [36]. The PHQ9 depression and suicidality (item 9) symptom scores were averaged, with higher scores denoting greater depression and suicidal ideation. Average scores across four IDASII domains (depression, traumatic symptoms (avoidance and intrusion), anxiety, and well-being) were obtained, with higher scores denoting greater depression, anxiety, traumatic symptoms, and well-being. The SASSR overall adjustment scores ranged from 0–5, with lower scores denoting higher social adjustment. All symptom measures (using the same instruments) were administered prior to the onset of the pandemic (*T1*) and during the pandemic (*T2*). 

### 2.7. Statistical Analysis

#### 2.7.1. Changes in Religious Importance (*RI*) and Religious Attendance (*RA*)

All statistical analyses were conducted in IBM SPSS (version 27, Armonk, NY USA) predictive analytics software. The changes in *RI* and *RA* from *T1* to *T2* in the overall sample were characterized using a pre- and post-repeated-measures design. First, the McNemar’s test for dependent samples was extended to the test of marginal homogeneity (*MH*) [37,38,39]. Building on these methods and guided by modern techniques [40,41], generalized linear mixed models (GLMM) were utilized to evaluate moderator effects. In the GLMM models, *RI* and *RA* were the target variables, modeled as ordinal-level data with a multinomial probability distribution and cumulative logit link function. An index variable, “time” was included to capture the two time points at data collection—*T1* and *T2*—and included a random intercept. We examined the fixed effects of gender (male vs. female)* time, age group (under 40 years vs. 40 years+)* time, educational level (less than college degree, vs. college degree/higher)* time, marital status (single/never married, married/remarried, separated/divorced/widowed)* time, MDD familial risk status (high risk vs. low risk)* time, and psychiatric history (no psychiatric history, past-only psychiatric history, recent psychiatric history* time) to establish whether the proportions of *RI* and *RA* differed from *T1* to *T2* across levels of these characteristics. Because age and generation were strongly correlated (*r* = −0.87), generation was not included among the fixed effects, since age captured much of the variance accounted for by generation. 

As a supplemental descriptor, and within each of the four *T1* categories of *RI* (not at all, slightly, moderately, highly) and *RA* (never, less than twice a year, about once a month, once a week or more), we examined the proportions in these categories at *T2*. We did not measure online religious attendance prior to the pandemic, so within the in-person *RA* categories, we examined the proportion of respondents who accessed online religious services at *T2*.

#### 2.7.2. Associations between *T1 RI*, *RA*, and Mental Health Outcomes at *T2*

Linear regressions based on the general linear model (GLM) were used to assess the systemic relationship between *T1* religiosity and post-pandemic mental health outcomes. The predictors were *RI* at *T1* and *RA* at *T1*; these two predictors were evaluated separately. The outcomes of interest were as follows: *T2* depression (PHQ-9 and IDAS-II); suicidality (PHQ-9 and IDASII), anxiety, traumatic symptoms, and well-being (DASII); and overall social adjustment (SASSR). Each outcome was evaluated separately. In each model, we controlled for demographics, which were age, gender, education level, and marital status. We also controlled for *T1* mental health symptoms. In addition, we examined moderation effects of religiosity and gender, age, MDD risk status, and psychiatric history. 

#### 2.7.3. Associations between *T2 RI*, *RA*, and Mental Health Outcomes at *T2*

We used GLM regression models to assess the systemic relationship between *RI* and *RA* (including online *RA*) during the pandemic (*T2*) and mental health outcomes in the same period. In addition to demographics and *T1* mental health symptoms, we also controlled for *RI* and *RA* at *T1*. We examined moderation effects of religiosity and gender, age, MDD-risk status, and psychiatric history.

#### 2.7.4. Supplemental Analyses: Association between Changes in *RI* and *RA* from *T1* to *T2* and Mental Health Outcomes during the Pandemic (*T2*)

Binary logistic regressions were used to evaluate the impact of change in religious importance and attendance on mental health outcomes. We compared decreases in *RI* or *RA* (vs. cases that remained the same/increased) from *T1*–*T2* in relation to demographic, clinical and COVID-19 experiential factors. We then compared effects of increases in *RI* or *RA* (vs. remained the same/decreased) from *T1*–*T2* on the same factors. 

The first predictor was decrease in *RI* and *RA* (referred to as no change or increase). The second predictor was increase in *RI* and *RA* (referred to as no change or decrease). We examined the effects of these changes on mental health outcomes as a function of demographics (gender, age, marital status, education), clinical risk factors (familial MDD risk, psychiatric history), and COVID-19-specific experiences. 

## 3. Results

### 3.1. Change in RI and RA from T1 to T2

#### 3.1.1. Change in Religious Importance (*RI*)

**Figure 2:**
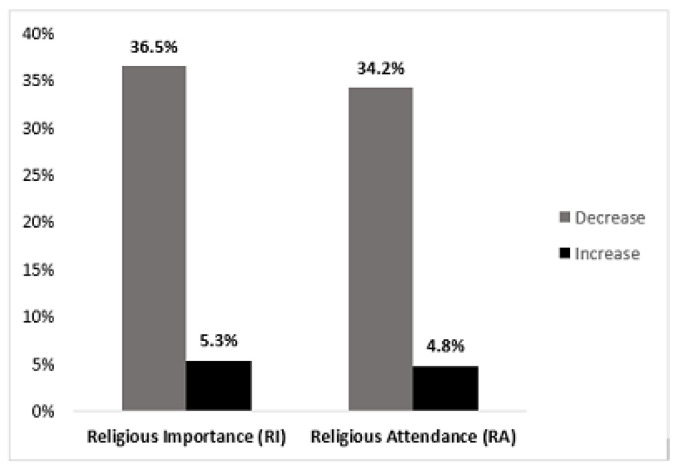
Changes in religious importance (*RI*) and religious attendance (*RA*) from *T1* to *T2.* Religious Importance (*RI*) *n* = 189; Religious Attendance (*RA*) *n* = 187. For *RI*, test of marginal homogeneity (*MH*) = 6.6, *p* < 0.001; for *RA*, test of marginal homogeneity (*MH*) = 5.8, *p* < 0.001.

The *RI* decreased for 69 individuals (36.5%), and increased for 10 individuals (5.3%), which was a significant difference based on the test of marginal homogeneity, (*MH*) = 6.6, *p* < 0.0001) (Figure 2). The changes in the proportions of *RI* from *T1* to *T2* did not differ significantly by gender (*p* = 0.64), age (*p* = 0.67), marital status (*p* = 0.77), educational level (*p* = 0.98), MDD risk status (*p* = 0.56), or psychiatric history (*p* = 0.99). 

As shown in Figure S1a and Table S1a (supplemental sections), before the pandemic (at *T1*), *RI* was distributed across the four categories as follows: not at all (*n* = 34, 18%), slightly (*n* = 47, 24.9%), moderately (*n* = 51, 30.2%), and highly (*n* = 51, 27.0%) important. After the pandemic’s onset (*T2*), the religious importance categories were distributed as follows: not at all (*n* = 52, 27.5%), slightly (*n* = 60, 31.7%), moderately (*n* = 46, 24.3%), and highly (*n* = 31, 16.4%) important. As indicated by the *MH* homogeneity test, there was significant movement out of the “highly important” category and into the “not at all important” category from *T1* to *T2*.

#### 3.1.2. Change in Religious Attendance (*RA*)

Overall, *RA* decreased for 64 individuals (34.2%) and increased for 9 individuals (4.8%) (Figure 2), yielding a significant difference between the proportion who decreased and the proportion who increased from *T1* to *T2* (*MH* = 5.8, *p* < 0.001). As shown in Figure S1b and Table S1b, the *T1 RA* was distributed as follows: never (*n* = 68, 36.4%), twice a year or less (*n* = 71, 38%), about once a month (*n* = 24, 12.8%), and once a week or more (*n* = 24, 12.8%). The *T2 RA* was distributed as follows: never (*n* = 107, 57.2%), twice a year or less (*n* = 54, 28.9%), about once a month (*n* = 8, 4.3%), and once a week or more (*n* = 18, 9.6%). Changes in *RA* from *T1* to *T2* did not differ significantly by gender (*p* = 0.71), age (*p* = 0.90), educational level (*p* = 0.67), marital status (*p* = 0.97), psychiatric history (*p* = 0.56), or MDD risk status (*p* = 0.62). As with the *RI*, and as indicated by the *MH* homogeneity test, there was significant movement out of the “once a week or more” category and into the “never” category.

The online religious attendance (Figure S1c, Table S1c) at *T2* was distributed across the following three categories: No (*n* = 157, 84%); yes, but less often than usual (*n* = 12, 6.4%); and yes, and as often as/more often than usual (*n* = 18, 9.6%). The “no” category was significantly larger than the other two categories (*χ*^2^ (2) = 215.94, *p* < 0.0001). The two “yes” categories did not differ significantly from one another (χ^2^ (1) = 1.20, *p* = 0.27). As with the *RI* and *RA*, these online religious attendance proportions were similar regardless of MDD-risk status, psychiatric history, gender, and age (data not shown but available upon request).

### 3.2. Effects of T1 RI and RA on Mental Health Outcomes during Pandemic (T2)

#### 3.2.1. Effects of *T1 RI* on *T2* Mental Health Outcomes

The T1 RI was significantly predictive of overall social adjustment (*B* = −0.10, *p* = 0.027), and marginally associated with lower suicidality (*B* = −0.20, *p* = 0.050) at *T2* (Table 1). The moderator effects (not shown in the tables or figures) are reported here. Gender moderated the relationship between *T1 RI* and IDASII well-being during the pandemic, *F* (1, 502.51) = 5.04, *p* = 0.03. The *T1 RI* among the women was associated with greater well-being during the pandemic, *B*= 0.77, s.e. (*B*) = 0.59, *p* = 0.19, and among men, it was associated with lower levels of wellbeing, *B* = −0.81, s.e. (*B*) = 0.56, *p* = 0.15. Psychiatric history moderated the relationship between *T1 RI* and *T2* IDASII depression, *F* (2, 273.07) = 3.43, *p* = 0.04. Among the respondents with a past psychiatric history, *T1 RI* predicted lower *T2* IDASII depression, *B* = −3.60, s.e. (*B*) = 0.94, *p* < 0.001. The other two categories (no psychiatric history and recent psychiatric history) were not significant. The MDD-risk status and age were not significant moderators of the relationship between *T1 RI* and mental health outcomes at *T2*.

#### 3.2.2. Effects of *T1 RA* on *T2* Mental Health Outcomes

Apart from a non-significant trend of *T1 RA* associated with traumatic symptoms at *T2*, we found no significant relationship between *T1 RA* and mental health outcomes at *T2* (Table 1). Psychiatric history moderated the relationship between *T1 RA* and IDASII depression during the pandemic, *F* (2, 286.42) = 3.61, *p* = 0.03. Among those with past-only psychiatric history, the *T1 RA* was associated with lower IDASII depression, *B* = −2.67, s.e. *(B*) = 0.96, *p* = 0.008. The other two categories (no psychiatric history and recent psychiatric history) were not significant. Gender and MDD-risk status were not significant moderators of the relationship between *T1 RA* and mental health outcomes at *T2*.

Age moderated the relationship between *T1 RA* and PHQ-9 depression, *F* (1, 72.85) = 4.95, *p* = 0.027, IDASII depression, *F* (1, 361.13) = 4.55, *p* = 0.034, IDASII traumatic symptoms, *F* (1, 24.06) = 4.21, *p* = 0.042, and SASSR overall social adjustment, *F* (1, 0.59) = 4.23, *p* = 0.041. Among the respondents aged 40+ years, *T1 RA* was significantly associated with greater overall social adjustment (SASSR), *B* = −0.10, s.e. (*B*) = 0.03, *p* = 0.01. Further, *T1 RA* was marginally associated with lower PHQ-9 depression, *B* = −0.53, s.e. (*B*) = 0.39, *p* = 0.18, and lower IDASII depression, *B* = −1.19, s.e. (*B*) = 0.73, *p* = 0.10. The *T1 RA* was not significantly associated with traumatic symptoms in this age group. Among those under 40 years of age, *T1 RA* was significantly associated with more traumatic symptoms at *T2*, *B* = 0.83, s.e. (*B*) = 0.29, *p* = 0.005, and marginally associated with higher PHQ-9 depression, *B*= 0.67, s.e. (*B*) = 0.43, *p* = 0.12 and higher IDASII depression, *B* = 1.5, s.e. (*B*) = 1.1, *p* = 0.20. The *T1 RA* was not significantly associated with overall social adjustment in this age group.

### 3.3. Effects of T2 RI and RA on Mental Health Outcomes during Pandemic (T2)

#### 3.3.1. Effects of *T2 RI* on *T2* Mental Health Outcomes

As shown in Table 2, T2 RI was associated with lower suicidality (PHQ-9), *B* = −0.06, *p* = 0.03. The moderator effects (not shown in tables or figures) are reported here. Psychiatric history moderated the relationship between *T2 RI* and IDASII depression during the pandemic *F* (2, 391.55) = 4.97, *p* = 0.008. Among those with a past-only psychiatric history, *T2 RI* was associated with lower depression, *B* = −3.21, s.e. (*B*) = 1.49, *p* = 0.036. Among those with no psychiatric history and recent psychiatric history, *T2 RI* was not significantly associated with depression.

Gender moderated the relationship between *T2 RI* and IDASII wellbeing at *T2, F* (1, 193.17) = 6.43, *p* = 0.012. Among the women, religious importance during the pandemic was marginally associated with greater wellbeing, *B* = 1.34, s.e. (*B*) = 0.94, *p* = 0.16. The MDD-risk status and age were not significant moderators of the relationships between *T2 RI* and mental health outcomes at *T2*.

#### 3.3.2. Effects of *T2 RA* on *T2* Mental Health Outcomes

The *RA* at *T2* was not associated with any mental health outcomes at *T2*. However, online religious attendance (Table 3) was significantly associated with lower IDASII depression (*B* = −2.35, *p* = 0.043) and anxiety (*B* = −0.72, *p* = 0.029). The relationships between *T2 RA* and mental health outcomes at *T2* were not significantly moderated by MDD-risk status, gender, age, or psychiatric history. The relationships between online religious attendance and mental health outcomes at *T2* were not significantly moderated by MDD risk status, gender, age, or psychiatric history.

### 3.4. Supplemental Analyses: Factors Associated with Changes in RI and RA from T1–T2

#### 3.4.1. COVID-19 Experiences and Decrease in *RI* and *RA* from *T1*–*T2*

Knowing someone who died from COVID-19 was the only factor significantly (and inversely) associated with a decrease in religiosity from waves 7–8 (*B* = −0.83, s.e. (*B*) = 0.40, *p* = 0.037). The individuals whose belief in religious importance decreased were less likely to know someone who had died of COVID-19, O.R. = 0.4, 95% confidence interval = (0.2–1.0) than those whose belief in religious importance remained the same or increased (Table S2a).

None of the demographic (age, education, marital status, gender), clinical (familial MDD risk, psychiatric history), or other COVID-19 experiential factors were significantly associated with decreases in religious attendance from *T1* to *T2* (Table S2b). However, there were non-significant trends among individuals with the following characteristics (Table S2b): in the females, *RA* was less likely to decrease than in the males; the individuals at high MDD risk were less likely than those at low MDD risk to present decreased *RA*; those with a college degree were more likely than those without a college degree to present decreased *RA*; and *RA* was less likely to decrease among those who reported feeling afraid and unsafe because of the COVID outbreak than among those who did not report feeling afraid or unsafe.

#### 3.4.2. COVID-19 Experiences and Increase in *RI* and *RA* from *T1*–*T2*

There were no significant associations between increases in *RI* from T1 to *T2* and demographic, clinical, or COVID-19 experiential factors (Table S3a). However, non-significant trends were noted among the individuals with the following characteristics: those younger than age 40 were more likely to present increased *RI* than those 40 or older; and those who reported that the pandemic made them feel unsafe or afraid were more likely to present increased *RI* than those who did not report feeling unsafe or afraid (Table S3a). Similarly, there were no significant associations between increases in *RA* from *T1*–*T2* and demographic, clinical, or COVID-19 experiential factors (Table S3b). However, there was a non-significant trend in which individuals at high MDD risk were less likely to increase their *RA* compared to those at low MDD risk.

## 4. Discussion

In this study, we aimed to investigate the relationship between the belief in the importance of religion and religious attendance and psychiatric symptoms and psychological wellbeing during the COVID-19 pandemic. Data on religious importance (*RI*) and attendance (*RA*), as well as data on psychiatric symptoms, were collected before (*T1*) and after the onset of the pandemic (*T2*), which minimized the artefacts from recall bias and retrospective reporting, which are affected by current emotional states. This is a significant strength, since most studies on religion and COVID-19 are limited by the use of data collected after the pandemic’s onset and their reliance on participants’ recall of pre-pandemic behaviors and symptoms.

Our results broadly indicate that belief in the importance of religion before the pandemic conferred some protection against depression and suicidality, and that it predicted overall social adjustment. Moreover, religious attendance online during the pandemic was associated with decreased depression and anxiety. This finding is consistent with those of other studies, which have shown general mental health benefits derived from religious beliefs and practices [10,12,42].

We found that most of the participants held the same beliefs regarding the importance of religion before and after the pandemic, although among the participants whose beliefs changed, a greater number showed a decrease than an increase in their belief in religious importance. In contrast to our findings, at least one previous study suggested that among those who held strong beliefs, beliefs were likely to increase during the pandemic [21]. Other studies have shown overall increases in religious practices, such as prayer or enhanced spirituality, during the pandemic [5,43]. The trajectories of religious beliefs before and during the pandemic appear to vary across countries, with increases in religiosity from pre- to post-pandemic onset occurring more frequently in the US than in other countries. These trajectories also appeared to vary across time, with religiosity in the US initially showing an increase in 2020 and early 2021, followed by a decrease during the latter phase of the pandemic, in late 2021 (Pew Research Center, 2021). Thus, our findings, which were based on a US sample during the earlier phase of the pandemic, were at odds with studies on religiosity during the same phase of the pandemic. Our study also found that the participants whose beliefs decreased were less likely to personally know someone who died from COVID-19. This absence of the personal impact of the pandemic amidst the global knowledge of the crisis may have challenged their existing religious beliefs and led to decreases in their faith. This is a relatively new finding and has not been replicated to date. Thus, this experience of the personal impact of the pandemic on religiosity may be an area of future research focus.

Similarly, as was the case for religious importance, we found that a greater proportion of those attending religious services before and after the pandemic continued to attend so or demonstrated decreased rather than increased attendance. Given the nature of the pandemic, a decrease in church/services attendance would be expected, and the desire to attend virtually could have been affected by Internet services and access, connectivity issues, and familiarity with the use of technology. However, our study demonstrated that virtual religious attendance during the pandemic had implications for mental health.

The relationships between religious importance and psychiatric outcomes were moderated by certain factors such as psychiatric history. It may be possible that relative to those without any psychiatric history or those with a recent psychiatric history, those with a past-only history of psychopathology were familiar with working strategies to prevent relapse, and that those who retained their beliefs in religious importance experienced less depression during the pandemic. A marginally significant effect of gender showed opposing effects of religious importance on psychological well-being for men and women. Religious importance prior to the pandemic was associated with greater wellbeing for women during the pandemic, but lower wellbeing for men. The reason for this discrepancy is unclear, but it may be related to the specific ways in which men and women use religious beliefs to cope with unpredictable crises [18,44]. This finding certainly warrants additional research, since studies on gender differences in religious coping have produced mixed results [45,46,47].

Religious attendance pre-pandemic did not seem to affect mental health overall during the pandemic, but certain clinical and demographic factors moderated this relationship. For example, among those with a past-only psychiatric history, religious attendance prior to the pandemic was predictive of lower depression during the pandemic. These individuals may have used religious attendance to cope with stress similarly to how they used religious beliefs; therefore, past experiences with religion were beneficial for them, but not for those without a psychiatric history or those with only a recent psychiatric history.

The individuals aged 40 years or older who attended religious services prior to the pandemic reported greater social adjustment than their younger counterparts during the pandemic. These older individuals who attended religious services prior to the pandemic also tended to display lower levels of depression than their younger counterparts. Among those under age 40, pre-pandemic religious attendance was associated with greater traumatic symptoms. Religious attendance may have been protective for older adults, but it was less so for younger adults, who may have sought religion only when faced with challenging circumstances and psychiatric symptoms.

During the pandemic, in-person religious attendance was marginally predictive of lower levels of depression, but online religious attendance was significantly predictive of lower depression and lower anxiety levels. While virtual meetings, services, and interactions have recently declined from their COVID-19 levels, many services continue to be provided online, either as a total replacement or as an adjunct for in-person modalities. This is also true of therapeutic medicine, including psychotherapy and psychoeducational groups. In turn, this has clinical implications, which are discussed further below.

*Clinical and Public Health Implications:* It is generally understood that individuals may rely on their faith during trying times, but acknowledgement of the decreased use of religion during global crises may pose a challenge for individuals who have relied on their faith in the past. Therefore, clinicians may need to tailor their therapeutic approaches around issues of disappointment, feelings of betrayal, growing distrust (in faith or in God) and survivor guilt, which may manifest among those not personally affected by the deaths of loved ones or acquaintances.

The apparent decrease in depression and anxiety among those who accessed religious services online bodes well for the effectiveness of accessible telemedicine. Additionally, telemedicine is a useful approach in situations that forbid in-person service delivery, or for individuals who have psychiatric disorders that prevent them from leaving home. Making virtual services accessible to the general public will be challenging, given the specific technological requirements and knowledge needed to safely access these services. The widespread availability and use of secure and HIPAA-compliant virtual health services is a critical public health issue.

*Limitations:* A careful consideration of the following limitations is advised in interpreting this study’s findings. First, the sample size was small and relatively homogenous. The small sample size reduced our power and ability to detect group differences. However, we were able to detect differences with larger effect sizes, which may be more clinically meaningful. Most of the respondents reported a Catholic religious affiliation, which had implications for their religious beliefs, their engagement in religious services, and the mental health benefits they derived from their religiosity. Studies have shown that religious denomination is a significant predictor of religious coping (Pew, 2022), and this may have been the case during the COVID-19 pandemic. This homogeneity restricted our ability to assess the participants’ beliefs and coping behaviors during the pandemic as a function of their religious affiliation. Second, and as stated in our introduction, religiosity and spirituality were used interchangeably in this study because of the way in which the questions were asked. In many contexts, these two terms can lead to different outcomes. However, the focus here was on assessing the extent to which reliance on a higher power was affected by the COVID pandemic, and how it affected mental health. Related to this limitation was the use of single items for religious beliefs and religious attendance, which may have limited our ability to parse out the effects of variations in religious beliefs and coping. Religious importance and religious attendance may not represent the spectrum of religious activities, which include prayer, reading the Bible, the relationships that arise from these activities, and so forth. Third, online religious attendance was assessed only after the pandemic’s onset, although our data on in-person religious attendance prior to the pandemic provided some insights about the effects of the pandemic on religious attendance, as well as its relationship with mental health.

## 5. Conclusions

Overall, our finding of the protective effects of religious beliefs and online religious attendance during the pandemic is very informative and lends itself to further inquiries into the possible mediators of religious beliefs and attendance (e.g., social support, prosocial behaviors, and characteristics).

Global crises can challenge existing religious beliefs. However, psychological benefits, such as increased wellbeing and decreased depression and suicidality, can be experienced by those who are able to utilize their beliefs and continue their practices. Many studies have distinguished between positive and negative religious coping—and further research might highlight the forms of religious coping that are challenged during events such as the COVID-19 pandemic. Given that relatively few of the respondents in our study reported an increase in their religious beliefs, our analyses revealed very little about the circumstances that are likely to be associated with such increases. Consequently, larger studies are needed to clarify the drivers of these religious trajectories. As expected, the nature of the pandemic forced the shutting down of institutions, places of worship, and venues of social interaction, leading to a decrease in religious attendance. During these periods, the individuals who found alternative ways of accessing religious services (e.g., online) when their traditional methods were absent or limited derived benefits, such as reduced anxiety and depression. In the same vein, therapeutic services can be delivered in similar modalities through a new public health focus on providing the psychiatric services to the public, while keeping individual privacy and confidentiality intact.

## Figures and Tables

**Table 1:** Effects of religious importance (***RI***) pre-pandemic (*T1*) on mental health outcomes during pandemic (*T2*).

MENTAL HEALTH OUTCOMES ^1^	Religious Importance (*RI*)		Religious Attendance (*RA*)	
	B (s.e. B)	*p*-Value	B (s.e.B)	*p*-Value
**PHQ-9**				
**Depression**	−0.08 (0.28)	0.777	0.19 (0.32)	0.541
**Suicidality**	0.01 (0.02)	0.721	−0.01 (0.02)	0.602
**IDAS-II**				
**Depression**	−0.22 (0.65)	0.734	0.15 (0.67)	0.823
**Suicidality**	−0.20 (0.10)	0.050 ^1^	−0.01 (0.11)	0.929
**Anxiety**	−0.14 (0.18)	0.462	0.04 (0.19)	0.821
**Traumatic Symptoms**	0.18 (0.18)	0.314	0.44 (0.18)	0.084 ^1^
**Well-Being**	−0.004 (0.41)	0.992	−0.31 (0.41)	0.458
**SASSR**				
**Overall Social Adjustment**	−0.10 (0.03)	0.027	−0.03 (0.03)	0.376

^1^ All GLM models control for demographic factors (gender, age, marital status, and education) *RI* and *RA* at *T2* and mental health outcomes at *T1*.

**Table 2:** Relationship between religious importance (*RI*) and religious attendance (*RA*) during pandemic (*T2*) and mental health outcomes at *T2*.

MENTAL HEALTH OUTCOMES ^1^	Religious Importance *(RI)*		Religious Attendance *(RA)*	
	B (s.e. B)	*p*-Value	B (s.e.B)	*p*-Value
**PHQ-9**				
Depression	0.15 (0.45)	0.738	−0.76 (0.43)	0.079 ^1^
Suicidality	**−0.06 (0.03)**	**0.027**	−0.02 (0.03)	0.569
**IDAS-II**				
Depression	−0.35 (1.05)	0.740	−1.67 (0.99)	0.095 ^1^
Suicidality	−0.002 (0.16)	0.989	−0.01 (0.16)	0.950
Anxiety	−0.39 (0.29)	0.191	−0.33 (0.28)	0.248
Traumatic Symptoms	0.17 (0.28)	0.560	−0.11 (0.27)	0.683
Well-Being	0.68 (0.65)	0.301	0.33 (0.62)	0.595
**SASSR**				
Overall Social Adjustment	−0.03 (0.04)	0.518	−0.05 (0.04)	0.208

^1^ All GLM models control for demographic factors (gender, age, marital status, and education) *RI, RA,* and mental health outcomes at *T1*.

**Table 3:** Relationship between online religious attendance (*T2*) and mental health outcomes at *T2*.

MENTAL HEALTH OUTCOMES ^1^	B (s.e. B)	*p*-Value
**PHQ-9**		
Depression	−0.76 (0.50)	0.133
Suicidality	−0.03 (0.03)	0.348
**IDAS-II**		
Depression	**−2.35 (1.16)**	**0.043**
Suicidality	−0.13 (0.18)	0.473
Anxiety	**−0.72 (0.33)**	**0.029**
Traumatic Symptoms	−0.52 (0.31)	0.097 ^1^
Well-Being	1.1 (0.73)	0.131
**SASSR**		
Overall Social Adjustment	−0.07 (0.05)	0.144

^1^ All GLM models control for demographic factors (gender, age, marital status, and education) *RI, RA,* and mental health outcomes at *T1*.

## Data Availability

Data supporting reported results can be obtained by writing to the corresponding author.

## Supplementary Materials

The following supporting information can be downloaded at: https://www.mdpi.com/article/10.3390/ijerph20116002/s1. Figure S1a: Changes in religious importance (*RI*) from *T1*–*T2* according to *T1* *RI* categories; Figure S1b: Changes in religious attendance (*RA*) from *T1*–*T2* according to *T1* *RA* categories; Figure S1c: Online religious attendance (*RA*) during pandemic (*T2*) according to *T1* in-person *RA* categories; Table S1a: Changes in religious Importance (*RI*) from *T1*–*T2* according to *T1* religious importance categories; Table S1b: Changes in religious attendance (*RA*) from *T1*–*T2*, according to *T1* religious attendance categories; Table S1c: Online religious attendance (*RA*) according to *T1* religious attendance categories; Table S2a: Effects of demographic, phenomenological, and clinical group on likelihood of decrease in *RI* from *T1*–*T2*; Table S2b: Effects of demographic, phenomenological, and clinical group on likelihood of decrease in *RA* from *T1*–*T2*; Table S3a: Effects of demographic, phenomenological, and clinical group on likelihood of increase in *RI* from *T1*–*T2*: Table S3b: Effects of demographic, phenomenological, and clinical group on likelihood of increase in *RA* from *T1*–*T2*.
