# Supplementary material for: Pre- and Post-Pandemic Religiosity and Mental Health Outcomes: A Prospective Study
Source: Int J Environ Res Public Health. 2023 May 30;20(11):6002. doi: 10.3390/ijerph20116002 (PMC10252618; doi:10.3390/ijerph20116002)
Supplement: Supplementary file 1 [file ijerph-20-06002-s001.zip › ijerph-2281286-supplementary.pdf]

Pre- and Post-Pandemic Religiosity and Mental Health Outcomes: A Prospective Study

SUPPLEMENTAL SECTION – Contents and pages:

Figure S1a: Changes in religious importance (*RI*) from *T1–T2* according to *T1 RI* categories (p. 2).

Figure S1b: Changes in religious attendance (*RA*) from *T1–T2* according to *T1 RA* categories (p.2).

Figure S1c: Online religious attendance (*RA*) during pandemic (*T2)* according to *T1* in-person *RA* categories (p.3).

Table S1a: Changes in religious Importance (*RI*) from *T1–T2* according to *T1* religious importance categories (p.4).

Table S1b: Changes in religious attendance (*RA*) from *T1–T2*, according to *T1* religious attendance categories (p.4).

Table S1c: Online religious attendance (*RA*) according to *T1* religious attendance categories. (p.4)

Table S2a: Effects of demographic, phenomenological, and clinical group on likelihood of decrease in *RI* from *T1–T2* (p.5).

Table S2b: Effects of demographic, phenomenological, and clinical group on likelihood of decrease in *RA* from *T1–T2* (p.6).

Table S3a: Effects of demographic, phenomenological, and clinical group on likelihood of increase in *RI* from *T1–T2* (p.7).

Table S3b: Effects of demographic, phenomenological, and clinical group on likelihood of increase in *RA* from *T1–T2* (p.8).

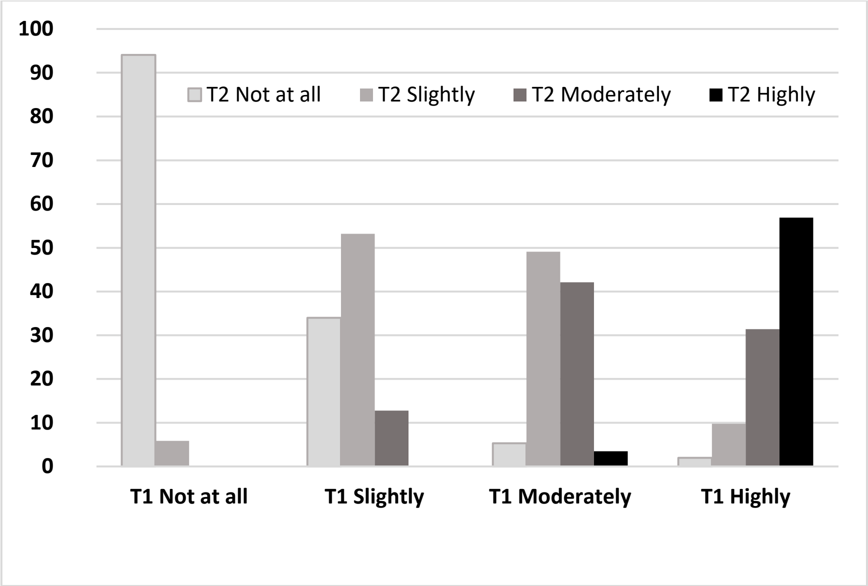

Figure S1a. Changes in religious importance (***RI***) from *T1-T2* according to *T1* ***RI*** categories. Note. For ***RI***, test of marginal homogeneity (MH) = 6.6, $p < 0.001$.

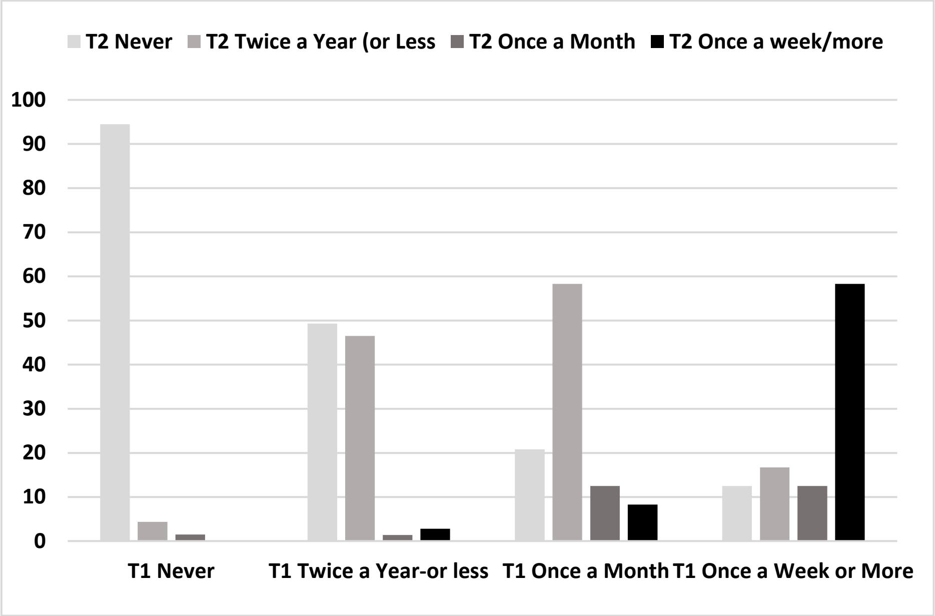

Figure S1b. Changes in religious attendance (***RA***) from *T1-T2* according to *T1* ***RA*** categories, Note. For ***RA***, test of marginal homogeneity (MH) = 5.8, $p < 0.001$.

.

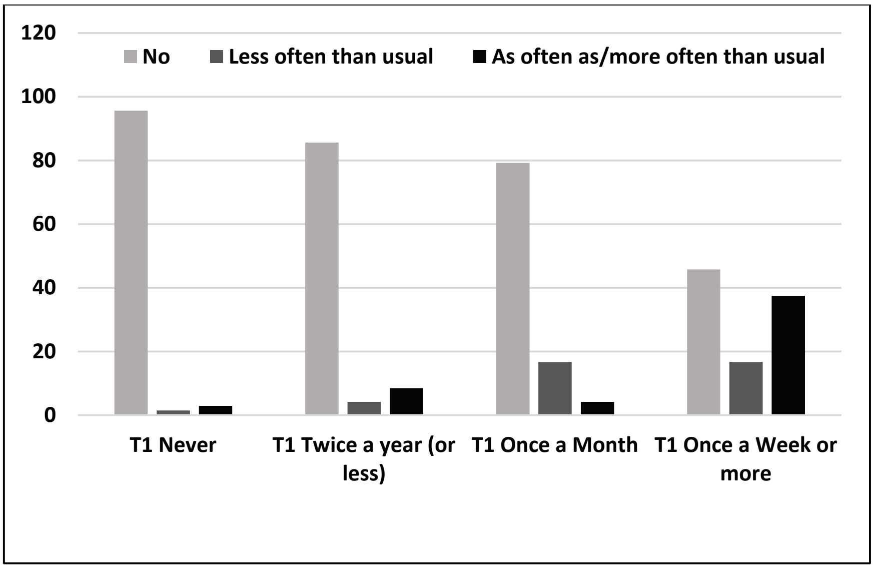

Figure S1c. Online religious attendance (***RA***) during pandemic (*T2)* according to *T1* in-person ***RA*** categories.

Table S1a. Changes in Religious Importance (***RI***) from *T1-T2* according to *T1* religious importance categories. Religious Importance (*n*=189)

Importance of Religion T1	Importance of Religion T2			
	Not at all (n=52)	Slightly (n=60)	Moderately (n=46)	Highly (n=31)
Not at all (*n*=34)	32 (94.1)	2 (5.9)	0	0
Slightly (*n*=47)	16 (34.0)	25 (53.2)	6 (12.8)	0
Moderately (n=51)	3 (5.3)	28 (49.1)	24 (42.1)	2 (3.5)
Highly (n=51)	1 (2.0)	5 (9.8)	16 (31.4)	29 (56.9)

Table S1b. Changes in Religious Attendance (***RA***) from *T1-T2*, according to T1 religious attendance categories. Religious Attendance (a) *n*=187

Religious Attendance T1	Religious Attendance T2			
	Never (n=107)	Twice a year/less (n=54)	Once a Month (n=8)	Once a week or more (n=18)
Never (*n*=68)	64 (94.1)	3 (4.4)	1 (1.5)	0
Twice a year (or less) (*n*=71)	35 (49.3)	33 (46.5)	1 (1.4)	2 (2.8)
Once a month (n=24)	5 (20.8)	14 (58.3)	3 (12.5)	2 (8.3)
Once a week/more) (n=24)	3 (12.5)	4 (16.7)	3 (12.5)	14 (58.3)

Table S1c. Online Religious Attendance (***RA***) according to *T1* religious attendance categories.

Religious *N* Attendance T1	Online Religious Attendance (During Pandemic)		
	No/Does not attend Religious Services (n=157)	Less often than usual (n=12)	As often/More often than usual (n=18)
Never (*n*=68)	65 (95.6)	1 (1.5)	2 (2.9)
Twice a year (or less) (*n*=71)	62 (85.6)	3 (4.2)	6 (8.5)
Once a month (n=24)	19 (79.2)	4 (16.7)	1 (4.2)
Once a week or more (n=24)	11 (49.8)	4 (16.7)	9 (37.5)

Note. Figures shown are N's (and percentages) within each row (T1 category) that endorse religious importance or attendance at T2. For religious importance, 15 cases deleted due to completion of T1 SADS after 3/2020 (7), and missing T1 SADS (8). **For religious attendance, 17 c**ases were deleted due to completion of T1 SADS after 3/2020 (7), missing T1 SADS (8), and missing data on religious attendance (2).

Table S2a. Effects of demographic, phenomenological and clinical group on likelihood to ***decrease RI*** from *T1-T2.*

FACTORS	B (s.e.)	*P*-value	Odds Ratio (95%-Confidence Interval)
DEMOGRAPHICS			
Female	0.34 (0.31)	.27	1.4 (0.8-2.6)
< Age 40 Years	-0.23 (0.30)	.45	0.8 (0.4-1.4)
Married	0.10 (0.33)	.74	1.1 (0.6-2.1)
Divorced/Widowed/Separated	-0.51 (0.51)	.31	0.6 (0.2-1.6)
College degree higher	0.10 (0.30)	.73	1.1 (0.6-2.0)
Hi MDD Risk	0.32 (0.31)	.30	1.4 (0.8-2.5)
Past (only) psychiatric history	0.44 (0.43)	.31	1.6 (0.7-3.6)
Recent Psychiatric history	0.09 (0.40)	.83	1.1 (0.5-2.4)
Getting tested for COVID-19	0.32 (0.31)	.29	1.4 (0.7-2.5)
Know someone hospitalized (With COVID-19)	0.09 (0.32)	.79	1.1 (0.6-2.1)
Know someone who died (from COVID-19)	**-0.83 (0.40)**	**.04**	**0.4 (0.2-1.0)**
COVID outbreak has made you feel unsafe/scared	-0.16 (0.33)	.63	0.9 (0.4-1.6)
Very/extremely satisfied with social support during pandemic	-0.19 (0.33)	.55	0.8 (0.4-1.6)
Communicated often/fairly often in while with family or friends since beginning of pandemic (Vs. never/once in a while)	-0.17	.64	0.8 (0.4-1.7)
Overall positive impact of pandemic on your and family	0.36 (0.51)	.48	1.4 (0.5-3.9)

Note. ***RI*** = belief in religious importance. Estimates obtained via binary logistic regression. Reference categories for demographic and clinical predictors are male, age 40+, married/remarried, less than college degree, low MDD risk, no psychiatric history. Reference categories for COVID experience predictors are "no." Reference Category for outcome (change in religious importance) is increased/stayed the same.

Table S2b. Effects of demographic, phenomenological and clinical group on likelihood to ***decrease* RA** from *T1-T2.*

FACTORS	B (s.e.)	*P*-value	Odds Ratio (95%-Confidence Interval)
DEMOGRAPHICS			
Female	-0.45 (0.31)	.15	0.6 (0.3-1.2)
< Age 40 Years	-0.01 (0.31)	.97	1.0 (0.5-1.8)
Divorced/Widowed/Separated	-0.21 (0.50)	.68	0.8 (0.3-2.2)
Married/remarried	-0.04 (0.34)	.91	1.0 (0.5-1.9)
College degree higher	0.47 (0.31)	.13	1.6 (0.9-2.9)
Hi MDD Risk	-0.53 (0.31)	.09	0.6 (0.3-1.1)
Past (only) psychiatric history	-0.34 (0.46)	.45	0.7 (0.3-1.7)
Recent Psychiatric history	0.33 (0.40)	.40	1.4 (0.6-3.1)
Getting tested for COVID-19	0.20 (0.31)	.51	1.2 (0.7-2.2)
Know someone hospitalized (With COVID-19)	0.35 (0.33)	.29	1.4 (0.7-2.7)
Know someone who died (from COVID-19)	0.14 (0.36)	.69	1.2 (0.6-2.3)
COVID outbreak has made you feel unsafe/scared	-0.58 (0.36)	.11	0.6 (0.3-1.1)
Very/extremely satisfied with social support during pandemic	-0.57 (0.23)	.70	0.9 (0.5-1.7)
Communicated often/fairly often in while with family or friends since beginning of pandemic (Vs. never/once in a while)	-0.43 (0.36)	.23	0.6 (0.3-1.3)
Overall positive impact of pandemic on your and family	0.34 (0.51)	.50	1.4 (0.5-3.8)

Note. **Note**. ***RA***= religious attendance. Estimates obtained via binary logistic regression. Reference categories for demographic and clinical predictors are male, age 40+, married/remarried, less than college degree, low MDD risk, no psychiatric history. Reference categories for COVID experience predictors are "no." Reference Category for outcome (change in religious importance) is increased/stayed the same.

Table S3a. Effects of demographic, phenomenological and clinical group on likelihood to *increase* ***RI*** from *T1-T2*

FACTORS	B (s.e.)	*P*-value	Odds Ratio (95%-Confidence Interval)
DEMOGRAPHICS			
Female	-0.73 (0.66)	.27	0.5 (0.1-1.8)
< Age 40 Years	1.0 (0.71)	.15	2.8 (0.7-11.0)
Divorced/Widowed/Separated			
Married/remarried			
College degree higher			
Hi MDD Risk	0.97 (0.81)	.23	2.6 (0.5-12.7
Past (only) psychiatric history	0.07 (0.94)	.93	1.1 (0.2-6.8)
Recent Psychiatric history	0.08 (0.86)	.94	1.1 (0.2-5.8)
Getting tested for COVID-19	0.31 (0.66)	.65	1.4 (0.4-5.0)
Know someone hospitalized (With COVID-19)	0.34 (0.67)	.61	1.4 (0.4-5.2)
Know someone who died (from COVID-19)	0.83 (0.67)	.21	2.3 (0.6-8.6)
COVID outbreak has made you feel unsafe/scared	0.92 (0.65)	.16	2.5 (0.7-9.0)
Very/extremely satisfied with social support during pandemic	-0.43 (0.67)	.52	0.6 (0.2-2.4)
Communicated often/fairly often in while with family or friends since beginning of pandemic (Vs. never/once in a while)	0.17 (0.81)	.83	1.2 (0.2-5.8)
Overall positive impact of pandemic on your and family	0.12 (0.86)	.89	1.1 (0.2-6.0)

Note. ***RI*** = belief in religious importance. Estimates obtained via binary logistic regression. Reference categories for demographic and clinical predictors are male, age 40+, married/remarried, less than college degree, low MDD risk, no psychiatric history. Reference categories for COVID experience predictors are "no." Reference Category for outcome (change in religious importance) is decreased/stayed the same.

Table S3b. Effects of demographic, phenomenological and clinical group on likelihood to ***increase* RA** from *T1-T2.*

FACTORS	B (s.e.)	*P*-value	Odds Ratio (95%-Confidence Interval)
DEMOGRAPHICS			
Female	0.42 (0.72)	.56	1.5 (0.4-3.2)
< Age 40 Years	-0.60 (0.72)	.40	0.5 (0.1-2.3)
Divorced/Widowed/Separated	1.01 (1.02)	.33	2.8 (0.4-20.6)
Married/remarried	0.63 (0.85)	.46	1.9 (0.4-10.0)
College degree higher	0.36 (0.69)	.60	1.4 (0.4-5.5)
Hi MDD Risk	-1.22 (0.72)	.09	0.3 (0.1-1.2)
Past (only) psychiatric history	N/A*	N/A	N/A
Recent Psychiatric history	N/A	N/A	N/A
Getting tested for COVID-19	0.60 (0.72)	.40	1.8 (0.4-7.5)
Know someone hospitalized (With COVID-19)	0.54 (0.69)	.44	1.7 (0.4-6.6)
Know someone who died (from COVID-19)	N/A	N/A	N/A
COVID outbreak has made you feel unsafe/scared	-1.25 (1.07)	.25	0.3 (0.03-2.3)
Very/extremely satisfied with social support during pandemic	1.33 (1.07)	.22	3.8 (0.5-30.8)
Communicated often/fairly often in while with family or friends since beginning of pandemic (Vs. never/once in a while)	0.90 (1.08)	.40	2.5 (0.3-20.3)
Overall positive impact of pandemic on your and family	0.10 (1.18)	.94	1.1 (0.1-11.2)

Note. ***RA***= religious attendance. Estimates obtained via binary logistic regression. Reference categories for demographic and clinical predictors are male, age 40+, married/remarried, less than college degree, low MDD risk, no psychiatric history. Reference categories for COVID experience predictors are "no." Reference Category for outcome (change in religious importance) is decreased/stayed the same.

*N/A means that there were zero or too few cases within a group to compute estimates.
